# Levodopa-Carbidopa Intestinal Gel in Parkinson's Disease: A Systematic Review and Meta-Analysis

**DOI:** 10.3389/fneur.2018.00620

**Published:** 2018-07-30

**Authors:** Libo Wang, Jia Li, Jiajun Chen

**Affiliations:** Department of Neurology, China-Japan Union Hospital of Jilin University, Changchun, China

**Keywords:** Parkinson's disease, levodopa-carbidopa intestinal gel, efficacy, safety, meta-analysis

## Abstract

**Background:** Levodopa has been widely used and regarded as the most effective therapy for Parkinson's disease (PD), but long-term treatment with oral levodopa may result in motor fluctuations and involuntary movements (dyskinesias). There is evidence to suggest that Continuous infusion of levodopa-carbidopa intestinal gel (LCIG) can effectively manage motor and non-motor complications in PD, but clinical studies investigating this have yielded inconsistent results. This systematic review and meta-analysis was performed to examine the efficacy and safety of LCIG for patients with PD.

**Methods:** A systematic search was conducted to retrieve published data in the EMBASE, PubMed, and the Cochrane Library up to March 2018. Both efficiency and safety of LCIG were analyzed using pooled standardized mean differences (SMDs) or odds ratio (ORs) with 95% confidence interval (CIs).

**Results:** Eight trials with 384 PD patients were included in the present study. Compared with the control group, LCIG significantly decreased off-time (SMD, −1.19; 95% CI, −2.25 to −0.12; *p* = 0.003) and increased on-time without troublesome dyskinesia (SMD, 0.55; 95% CI, 0.20 to 0.90; *p* = 0.002). However, no significant difference of LCIG was found in on-time with troublesome dyskinesia. There were no significant differences in UPDRS, Hoehn & Yahr and PDQ-39 scores. Besides, no significant differences in the drop-out and adverse effects.

**Conclusions:** Continuous delivery of LCIG may offer a promising option for PD patients. More randomized double-blind controlled studies with large sample sizes were needed to further confirm the efficacy and safety of LCIG for PD patients.

## Introduction

Parkinson's disease (PD) is the second most common chronic neurodegenerative diseases characterized by resting tremor, bradykinesia, rigidity, and postural instability. About 1% of the subjects aged over 60 years suffer from PD worldwide (1, 2). According to the survey from National Parkinson's Foundation in 2010, there were ~1 million patients suffering from PD and 50,000 to 60,000 new cases that are diagnosed with PD every year in the USA. The prevalence of PD for people aged over 65 years was 1.7% in China (3) and it is predicted to increase because of the population aging.

Levodopa is the amino-acid precursor of dopamine and replenishes the depleted striatal dopamine. Since more than 40 years, levodopa has been widely used as the most effective treatment for advanced Parkinson's disease (4–6). However, due to its short plasma half-life, oral levodopa may cause pulsatile striatal receptor stimulation, and thereby lead to dyskinesias, troublesome motor fluctuations, and unpredictable swings from mobility to immobility (7, 8). Levodopa-carbidopa intestinal gel (LCIG) was developed by using a percutaneous pump and helps to offer more continuous dopaminergic stimulation and leads to more constant plasma levels of levodopa (9). Clinical evidence suggests that LCIG results in significant decrease in off-time and on-time with troublesome dyskinesias and motor fluctuations (10–12). Surgical- or device-related complications are the most common side effects, but there are also anecdotal reports on neuropathy in LCIG-treated patients (13).

In recent years, increasing evidence has described the pharmacokinetics (14–16), efficacy and safety after LCIG treatment in patients with PD (12–14, 17–27). However, these results are variable and controversial. No clear consensus has been reached on the efficacy and safety of LCIG. Therefore, we performed this systematic review and meta-analysis to investigate the efficacy, safety, and tolerability of LCIG for PD patients based on the reported evidence.

## Materials and methods

### Search strategy

A comprehensive literature search was performed to collect the potential reports in electronic databases PubMed, Embase, and the Cochrane Library up to March 2018. The reference lists of retrieved studies were also reviewed and identified. Both subject terms and free terms were used in the search progress, including “Parkinson disease,” “Parkinson's,” “carbidopa,” “levodopa,” “gel,” and “intestines.” No study types were restricted during the search. No language restrictions were set.

### Eligibility criteria

Studies were eligible when they met the following entry criteria: (1) patients with Parkinson's disease; (2) exposure to LCIG; (3) studies with a control group; (4) sample size > 10; (5) reported the efficacy and/or safety of LCIG after treatment. No sex and ethnicity of patients were restricted. These eligibility criteria were verified based on the search results.

### Data extraction

Two review authors independently performed the data extraction from included trials. The information included (1) the first author and publication date, (2) study design, (3) treatment arms, (4) the country where the study conducted, (5) sample size and the sex ratio, (6) treatment duration, (7) outcomes of interest, and (8) main findings of each selected trial. For the outcomes, we further collected the following parameters: on time, off time, UPDRS, Hoehn & Yahr, PDQ-39, and adverse effects. The disagreement between two reviewers was resolved by discussion with another author.

### Quality assessment

Two authors evaluated the quality of each study included in this meta-analysis. The standard scoring criteria proposed by the Cochrane Back Review Group (28) were used for the four RCTs, and the Newcastle-Ottawa Quality Assessment Form (29) was used to for the other four cohort studies. The scoring criteria for RCTs included five domains, i.e., (1) selection bias, (2) performance bias, (3) attrition bias, (4) reporting bias, and (5) outcome assessor blinding and timing of outcome assessment. There are 12 items which scored a total of 12 points. A study scored more than 8 points was regarded as a high-quality study. A study scored fewer than 5 points was regarded as a low-quality study. The Other studies can be regarded as moderate-quality studies.

The Newcastle-Ottawa Quality Assessment Form included three domains, i.e., selection (4 points), comparability (2 points), and outcome (3 points). There are 12 items which scored a total of 12 points. A high-quality study scored more than 7, whereas a low-quality study scored less than 6. Other studies scored 6–7 were rated as a moderate grade. Uncertainty or disagreement was resolved by discussion to reach a consensus.

### Outcome measures

This meta-analysis focused on the efficacy and safety of LCIG for PD. The primary efficacy outcomes were the changes in ON time and OFF time; secondary endpoints were the changes in UPDRS, Hoehn & Yahr, and PDQ-39. Safety outcomes included the incidences of adverse event (AE) and serious AEs, and treatment withdrawal for any reasons and for AEs. Other parameters of pharmacokinetics and laboratory tests were narratively reviewed.

### Statistical analysis

Chi-squared test and the *I*^2^ statistics were used for the assessment of heterogeneity among the included studies (2, 30, 31). When *I*^2^ ≥ 50% or *p* ≤ 0.1, there was a significant heterogeneity and a random-effects model was utilized. Otherwise, a fixed-effects model was adopted when *I*^2^ < 50%. For continuous variables, standardized mean differences (SMDs), which are more suitable than weighted mean differences (WMDs) when the mean differences were large across studies (32), and the 95% confidence intervals (CIs) were used. For dichotomous outcomes, pooled odds ratios (ORs) and corresponding 95% CIs were calculated for the measurement of differences compared with control groups. Data analysis was performed using a Review Manager software, version 5.3 for Windows (Cochrane Collaboration, Oxford, United Kingdom). Reported probability values were two-sided, with significance set at *p* ≤ 0.05. We did not perform the sensitivity analyses because of the limited number of eligible articles (33).

## Results

### Study selection

**Figure 1:**
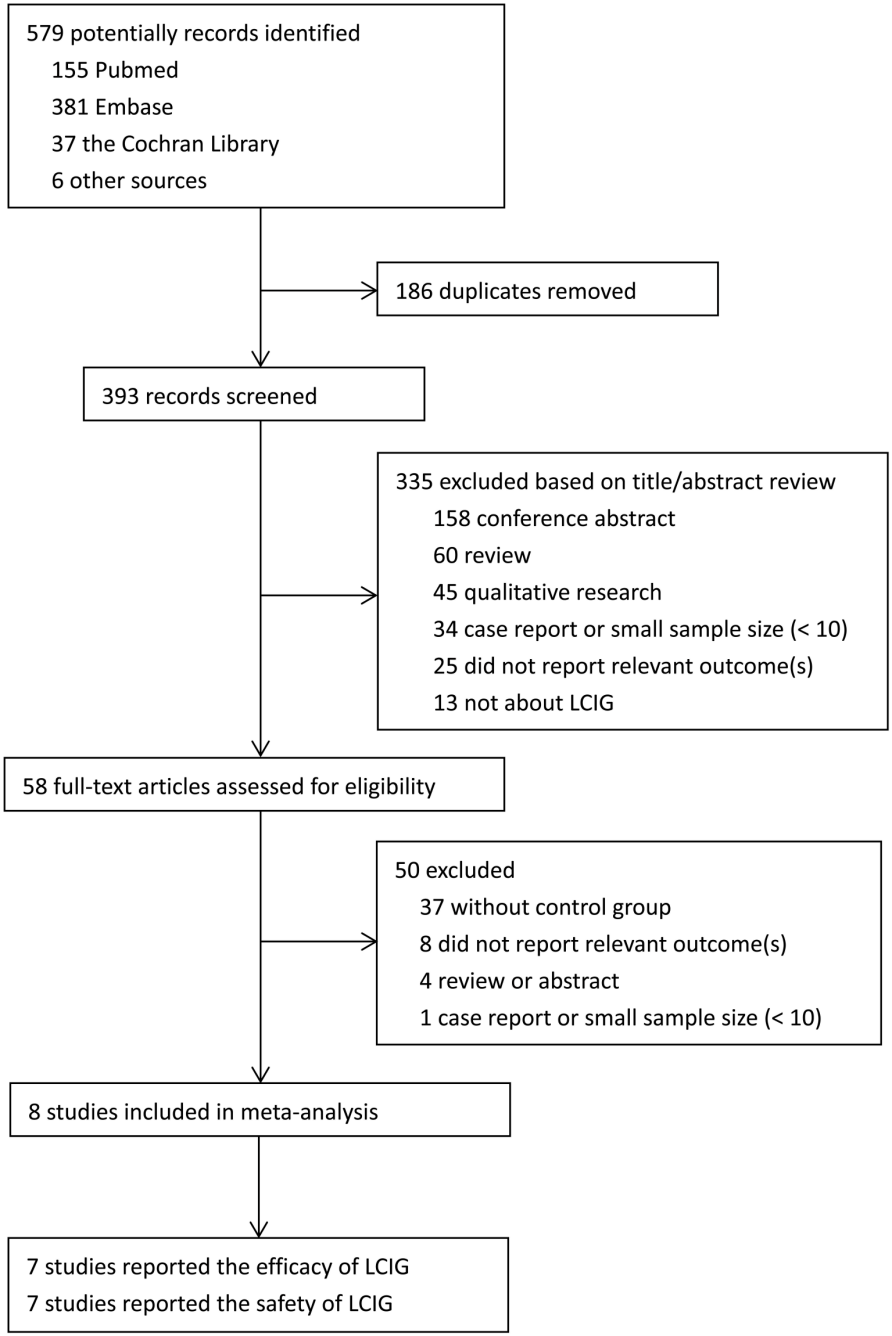
PRISMA flow chart of Study Selection in Meta-analysis.

Literature selection process was performed as shown in the PRISMA flow diagram (Figure 1). Briefly, a total of 579 studies were collected from various electronic databases and the reference lists of retrieved studies. Then EndNote X7 software was used for the removal of duplicated studies. The potential studies were screened by two authors based on the title/abstract. They further identified the selected studies by reading the full text. The disagreements were discussed with a third author to reach a consensus. Finally, eight trials with 384 Parkinson's disease patients were included in the present meta-analysis (12, 14, 17, 18, 21, 23, 24, 27). Among them, seven studies reported the efficacy of LCIG (12, 14, 17, 18, 21, 23, 24), and seven studies reported the safety of LCIG (12, 14, 17, 21, 23, 24, 27).

### Study characteristics

The characteristics of included trials were shown in Table 1. All studies were RCTs (12, 14, 17, 21) or cohorts (18, 23, 24, 27) published between 2003 and 2017. Most studies compared the LCIG with oral medical treatment (12, 14, 17, 18, 21, 23), while one study compared the LCIG with subthalamic nucleus deep brain stimulation. Three of the studies were performed in Sweden (14, 17, 24), two in USA (12, 21), one in Germany (18), one in Spain (27), and one in both USA and Italy (23). All patients were suffered from advanced PD and were treated with LCIG. Interestingly, there were a lot more male patients than female patients in most included studies. The treatment duration was varied from 3 weeks to 5 years. Besides the efficacy and safety of LCIG, some included studies also reported the pharmacokinetics changes. Most included studies showed that LCIG was a safe therapeutic option for PD patients.

**Table 1:** Characteristics of eight included studies.

**Study (year)**	**Design**	**Arms**	**Country**	**Population (N, age)**	**Sex: Male/Female**	**Follow-up duration (months)**	**Main outcome measures**	**Findings**	Risk of bias^*^
Nyholm et al. (9)	RCT	Intestinal infusion (L/C: 50/12.5 mg), OMT (L/C: 200/50 mg)	Sweden	*N* = 12, 61.2 ± 11.0 years old	10/2	3 weeks	Pharmacokinetics, efficacy outcome	Continuous intraduodenal delivery of a new carbidopa/levodopa formulation offers a means for markedly improved control of motor fluctuations in late stages of PD.	7 (moderate)
Nyholm et al. (17)	RCT	Duodenal levodopa infusion monotherapy (L/C: 20/5 mg), OMT (L/C: 20/5 mg)	Sweden	*N* = 45, 50–79 years old	18/6	6 months	UPDRS score, AE	Continuous intraduodenal infusion of the LCIG as monotherapy is safe and clinically superior to a number of individually optimized combinations of conventional oral and subcutaneous medications in patients with motor fluctuations.	8 (moderate)
Jugel et al. (18)	Prospective cohort	LCIG (1961 ± 640 mg), OMT (1526 ± 520 mg)	Germany	*N* = 30, 69 ± 8 years old	20/10	736 ± 420 days	Sensory and motor assessments, correlations	The results are compatible with the promotion of axonal neuropathy by LCIG infusion.	5 (low)
Olanow et al. (12)	RCT	LCIG (91.7 ± 96·6 mg), OMT (249.7± 94.9 mg)	USA	*N* = 71, 64.4 ± 8.2 years old	46/25	12 weeks	Efficacy outcome, AE	LCIG provides a therapeutic option for patients with advanced Parkinson's disease who have off-episodes that cannot be satisfactorily controlled with standard medical therapies.	10 (high)
Slevin et al. (21)	RCT	LCIG (L/C: 20/5 mg/mL), LC-IR (L/C: 20/5 mg/mL)	USA	*N* = 62, 64.1 ± 7.9 years old	44/18	52 weeks	Efficacy outcome, AE, QoL	Continuing-LCIG patients continued to derive benefit from LCIG while the magnitude of improvement among LCIG-naïve patients was similar to that observed for patients on LCIG in the preceding double-blind study. The overall AE profile was consistent with previous phase 3 clinical trials involving the LCIG system.	9 (high)
Merola et al. (23)	Retrospective cohort	LCIG (1272 ± 432 mg), OMT (1205 ± 421 mg), STN-DBS (1383 ± 458 mg)	Italy & USA	*N* = 40, 46–69 years old	NP	5 years	Efficacy outcome, AE, activities of daily living, motor complications	STN-DBS and LCIG showed comparable efficacy in ADL and OFF time, superior to OMT. STN-DBS yielded greater improvement in dyskinesia and lower long-term rate of complications than LCIG.	7 (moderate)
Palhagen et al. (24)	Prospective cohort	LCIG (1376 ± 496 mg), LCIG-naïve (1784 ± 724 mg)	Sweden	*N* = 77, 65.4 ± 5.2 years old	NP	3 years	Efficacy outcome, AE	LCIG treatment led to significant improvements in motor function and QoL over 18 months in LCIG-naïve patients and no worsening was observed in LCIG-experienced patients over 3 years despite natural PD progression over time.	6 (moderate)
Valldeoriola et al. (27)	Prospective cohort	LCIG (1,145 ± 305 mg), STN-DBS (900 ± 275 mg)	Spain	*N* = 47, 51–72 years old	38/9	12 months	Motor assessments, AE	Patients treated with LCIG may significantly improve some specific neuropsychological functions when compared with patients receiving STN-DBS and with patients receiving conventional medical treatment after 1 year from the intervention; there are not significant cognitive or behavioral changes in patients treated with STN-DBS when compared to PD patients receiving conventional medical treatment after 1 year from the intervention.	7 (moderate)

* *Cohort studies were assessed by the Newcastle-Ottawa Quality Assessment Scale. RCTs were assessed by the standard scoring criteria proposed by the Cochrane Back Review Group. The scores are presented as the total score. RCT, randomized controlled trial; L/C, levodopa/carbidopa; PD, Parkinson's disease; LCIG, levodopacarbidopa intestinal gel; OMT, oral medical treatment; UPDRS, Unified Parkinson's Disease Rating Scale; AE, adverse effects; LC-IR, levodopa-carbidopa immediate release; STN-DBS, subthalamic nucleus deep brain stimulation; ADL, activities of daily living; NP, not reported; QoL, quality of life*.

### Study quality

In general, a randomized controlled trial (RCT) has higher quality compared with an observational study. Study quality of RCTs was evaluated by using an assessment scale proposed by the Cochrane Back Review Group, and the results of risk of bias were presented in Table 1. Two RCTs were classified as high quality (12, 21), and the other two RCTs were classified as moderate quality (14, 17). The qualified trials scored ranging from 7 to 10. For the other four cohort studies evaluated by Newcastle-Ottawa Quality Assessment Form, two studies (23, 27) scored 7 points and one study (24) scored 6 points, which could be regarded as at moderate-quality. While one study only (18) scored 5 points, and could be regarded as at low-quality. Thus, most included studies were deemed to be of the moderate or high quality, except one (18). Most RCTs lost points because of the lack of blinding and adequate random sequence generation. While most cohort studies lost points because of a statement of the outcome of interest at the beginning and non-blind outcome assessment.

### On-time and off-time

**Figure 2:**
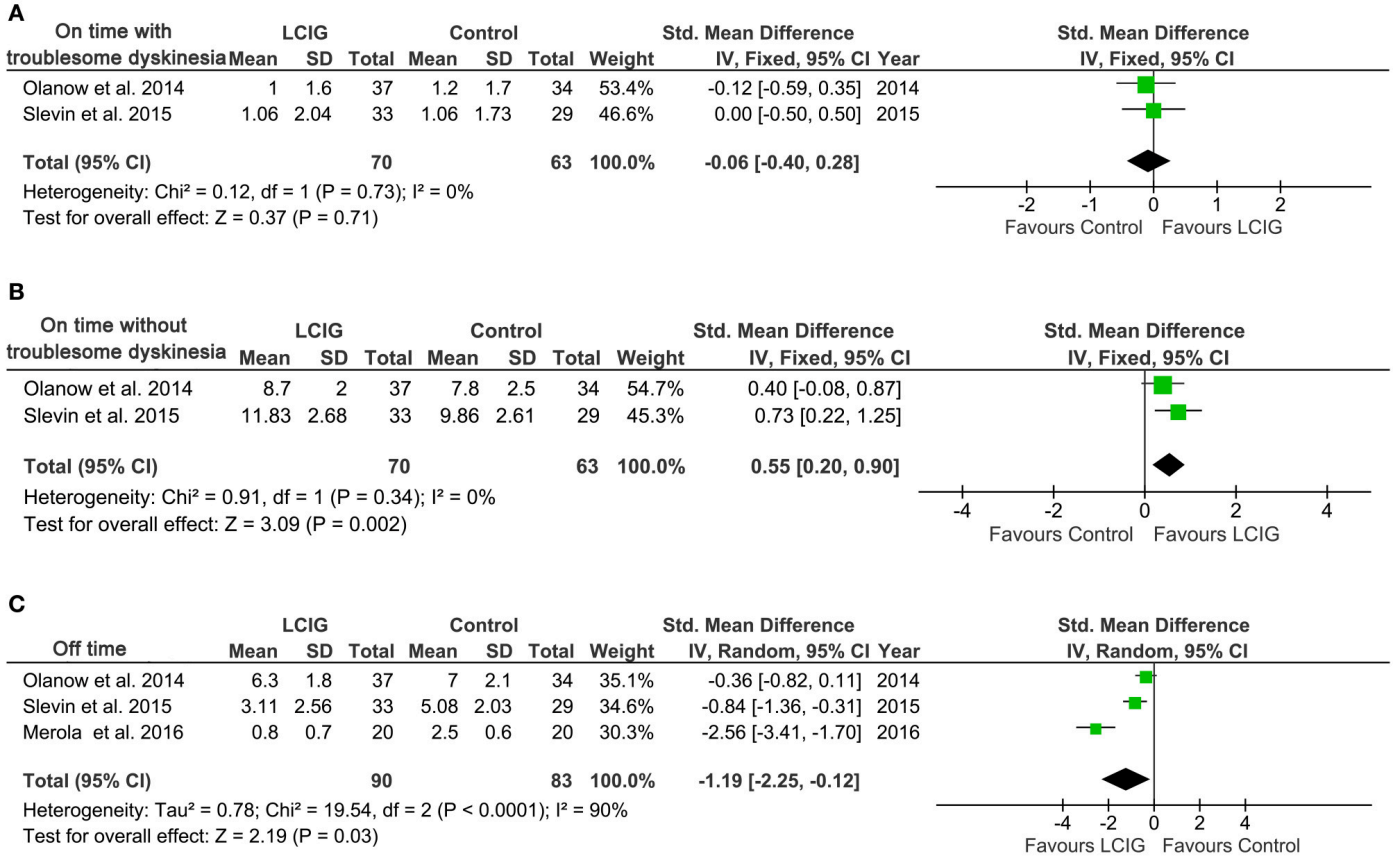
Forest plots of pooled on-time and off-time in PD patients. Plot A **(A)**, on-time with troublesome dyskinesia, h per day; plot B **(B)**, on-time without troublesome dyskinesia, h per day; plot C **(C)**, off-time, h per day. PD, Parkinson's disease; LCIG, levodopa-carbidopa intestinal gel.

Two studies reported on-time with/without troublesome dyskinesia (12, 21), involving 133 PD patients following LCIG. While three studies reported the off-time (12, 21, 23), involving 173 PD patients following LCIG. As shown in Figure 2, no notable heterogeneity was found in on-time with/without troublesome dyskinesia (*I*^2^ = 0%, *p* = 0.73; *I*^2^ = 0%, *p* = 0.34), while a notable heterogeneity was found in off-time (*I*^2^ = 90%, *p* < 0.0001). There was no significant difference in on-time with troublesome dyskinesia (SMD, −0.06; 95% CI, −0.40 to 0.28; *p* = 0.71) between control and LCIG group (Figure 2A). However, Significant differences were found in on-time without troublesome dyskinesia (SMD, 0.55; 95% CI, 0.20 to 0.90; *p* = 0.002; Figure 2B) and off-time (SMD, −1.19; 95% CI, −2.25 to −0.12; *p* = 0.003; Figure 2C), which suggested the positive effects of LCIG.

### UPDRS

**Figure 3:**
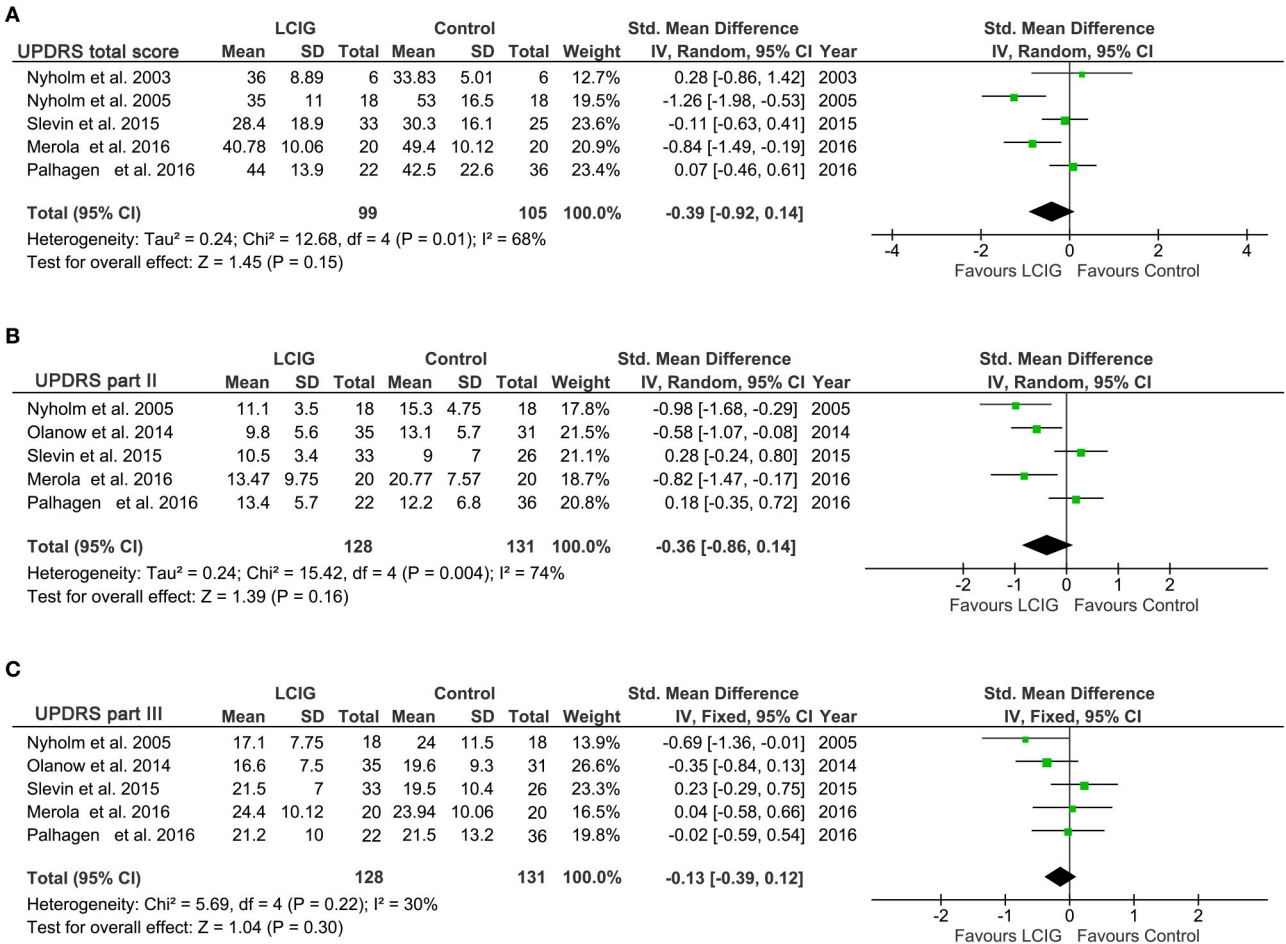
Forest plots of pooled UPDRS scores in PD patients. Plot A **(A)**, UPDRS total scores; plot B **(B)**, UPDRS part II; plot C **(C)**, UPDRS part III. PD, Parkinson's disease; LCIG, levodopa-carbidopa intestinal gel.

For UPDRS, five studies with 204 PD patients reported UPDRS total score (14, 17, 21, 23, 24) and other five studies with 259 PD patients reported UPDRS part II and part III (12, 17, 21, 23, 24). A notable heterogeneity was found in UPDRS total score (*I*^2^ = 68%, *p* = 0.01) and UPDRS part II (*I*^2^ = 74%, *p* = 0.004), while no significant heterogeneity was found in UPDRS part III (*I*^2^ = 30%, *p* = 0.22). Therefore, a random-effects model was used for UPDRS total score and UPDRS part ?, while a fixed-effects model was used for UPDRS part II. The pooled results showed that LCIG did not significantly improve UPDRS total score and UPDRS part II and part III (all *p* > 0.05; Figure 3).

### PDQ-39

**Figure 4:**
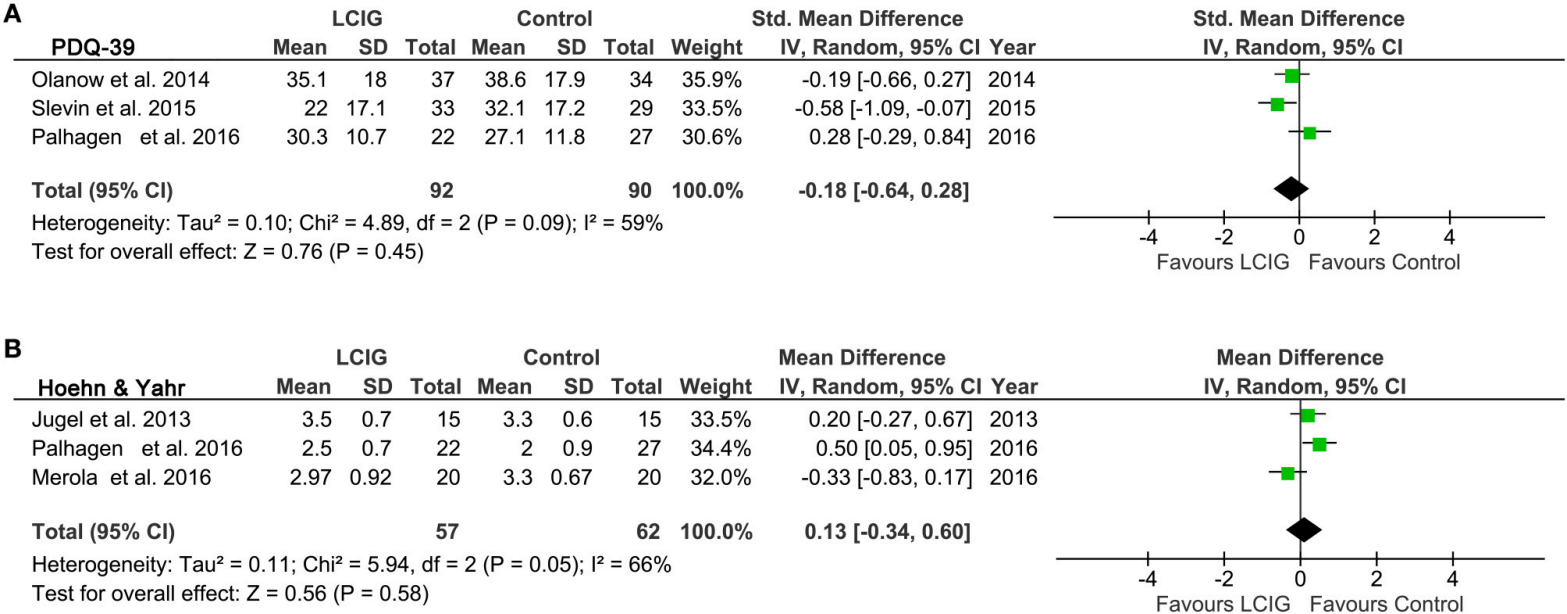
Forest plots of pooled PDQ-39 and Hoehn & Yahr scores in PD patients. Plot A **(A)**, PDQ-39 scores; plot B **(B)**, Hoehn & Yahr scores. PD, Parkinson's disease; LCIG, levodopa-carbidopa intestinal gel; PDQ-39, Unified Parkinson's Disease Rating Scale.

We analyzed PDQ-39 from three studies with 182 patients. The comparisons of PDQ-39 presented a significant heterogeneity among the studies, as was evident from *I*^2^ = 59%, *p* = 0.09 (Figure 4A). Thus, a random-effects model was adopted in this comparisons. Pooling trials that reported PDQ-39 got an SMD of −0.18 and 95% CI of −0.64 to 0.28, which was not a significant effect in favor of LCIG (*p* = 0.45).

### Hoehn & yahr

Three studies with 119 patients reported the outcome of Hoehn & Yahr. The heterogeneity is significant, as was evident from *I*^2^ = 66%, *p* = 0.05 (Figure 4B). Pooled results using a random-effects model showed that Hoehn & Yahr got an SMD of 0.13 and 95% CI of −0.34 to 0.60, which was also not a significant effect in favor of LCIG (*p* = 0.58).

### Tolerability

**Figure 5:**
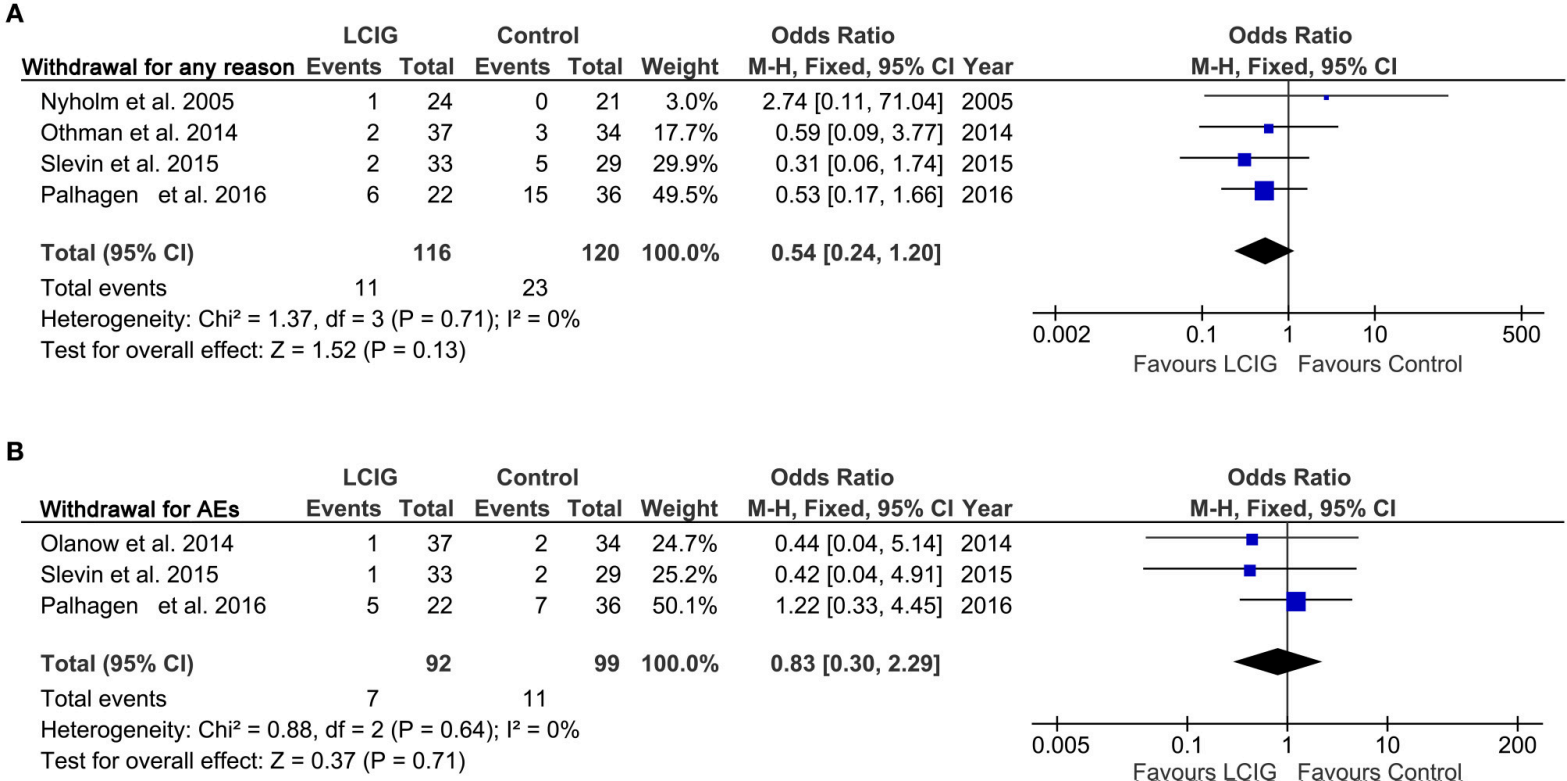
Forest plots of pooled withdrawal in PD patients. Plot A **(A)**, withdrawal for any reason; plot B **(B)**, withdrawal for AEs. PD, Parkinson's disease; AEs, adverse effects; LCIG, levodopa-carbidopa intestinal gel.

Treatment withdrawal (12, 17, 21, 24) were discussed in four studies for any reason. Among them, three trials discussed the treatment withdrawal for adverse effects (AEs) (12, 21, 24). No significant heterogeneity was observed, and thus, a fixed effect model was adopted when analyzed the treatment withdrawal. No significant difference was found between LCIG and control groups in regard to dropouts (OR, 0.54; 95% CI, 0.24 to 1.20; *p* = 0.13; Figure 5A; OR, 0.83; 95% CI, 0.30 to 2.29; *p* = 0.71; Figure 5B).

### Adverse effects (AEs)

**Figure 6:**
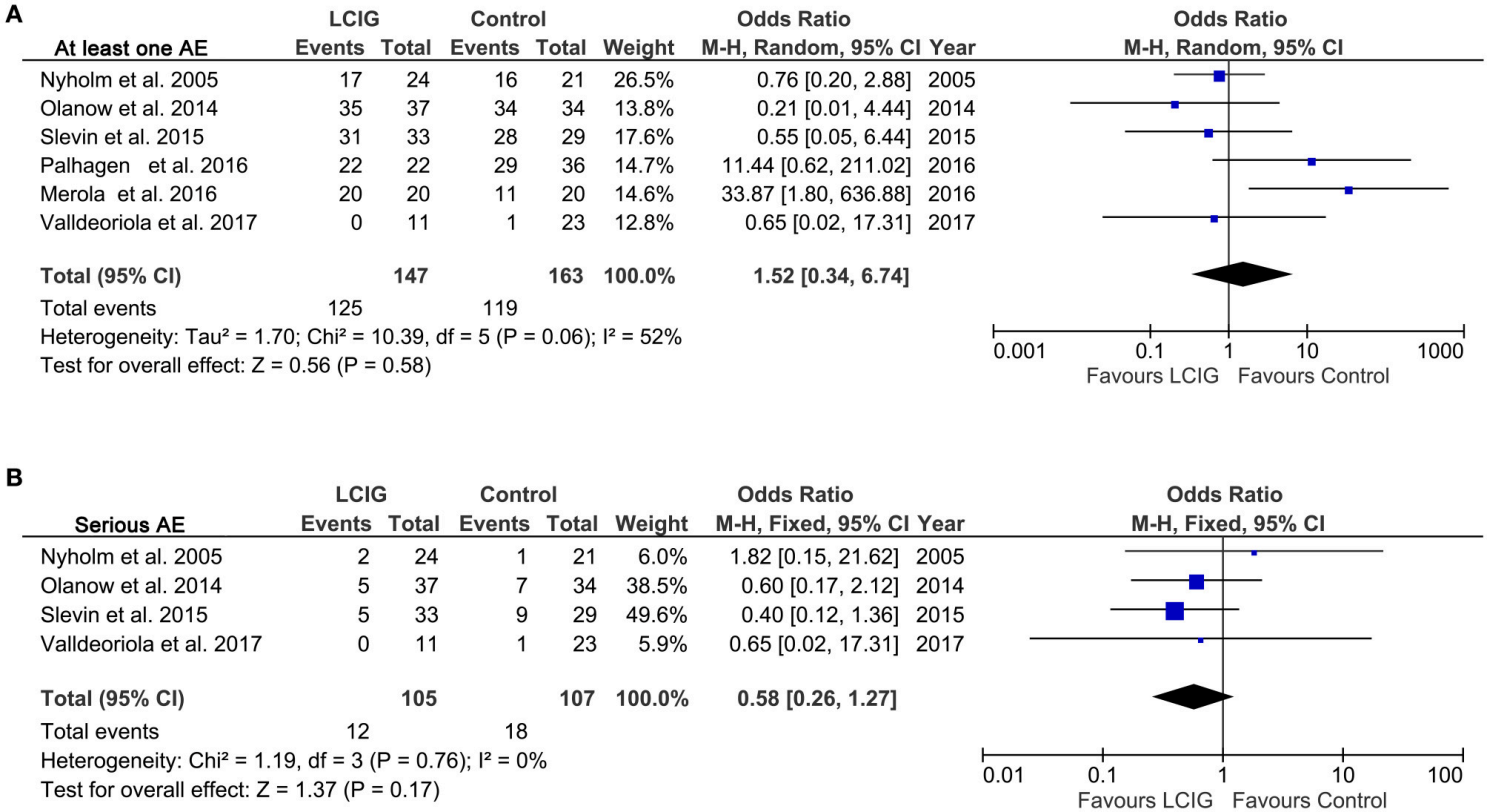
Forest plots of pooled AE in PD patients. Plot A **(A)**, at least one AE; plot B **(B)**, serious AE. PD, Parkinson's disease; LCIG, levodopa-carbidopa intestinal gel; AE, adverse effect.

Various AEs were discussed in most included studies. We performed meta-analysis according to the comparison of at least one AE and serious AE. A notable heterogeneity was found in AE (*I*^2^ = 52%, *p* = 0.06), while no notable heterogeneity was found in serious AE (*I*^2^ = 0%, *p* = 0.76). No meaningful differences were found in the risk of AEs (OR, 1.52; 95% CI, 0.34 to 6.74; *p* = 0.58; Figure 6A) and severe AEs (OR, 0.58; 95% CI, 0.26 to 1.27; *p* = 0.178; Figure 6B) between control and LCIG. No deaths were reported in the control or LCIG groups of most included studies. However, four deaths occurred in one study (24) (control, *n* = 2 and LCIG, *n* = 2). The relationship to study drug was classified by a local study investigator to be unrelated (*n* = 2), unlikely related (*n* = 1) to medications and possibly related (*n* = 1; cardiac arrest) (24).

### Publication bias

**Figure 7:**
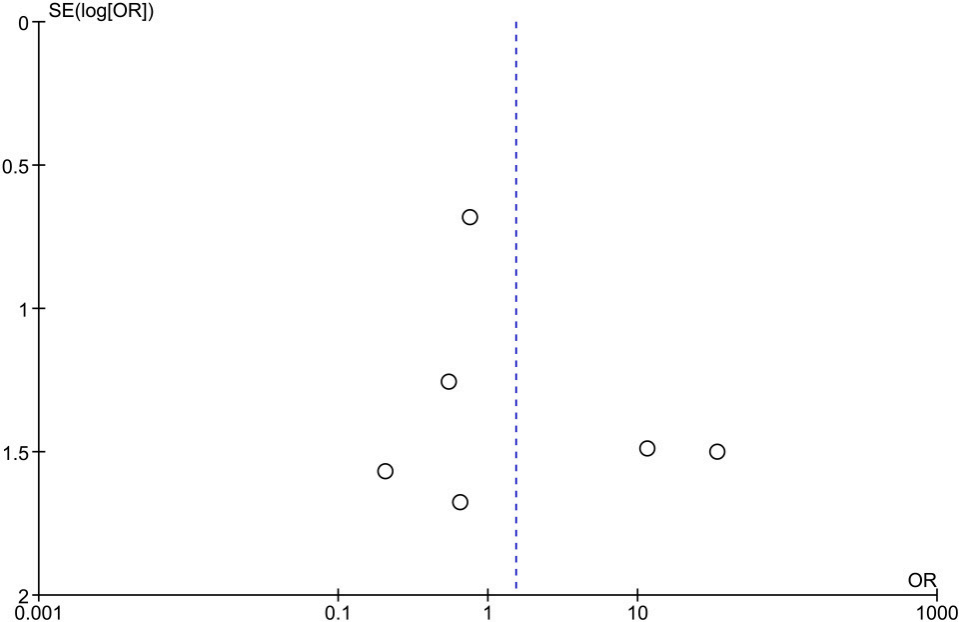
Funnel plot of comparison for at least one AE. The funnel plot appeared asymmetric. Each small circle represents an independent study for the indicated association. AE, adverse effects.

Publication bias was estimated by funnel plots. No obvious asymmetry was identified in funnel plots of all outcomes except at least one AE. The funnel plot was quite asymmetrical, suggesting that the publication bias of at least one AE should not be ignored (Figure 7).

## Discussion

The current systematic review and meta-analysis provide evidence that continuous infusion of LCIG provided a clinically significant increase in on-time without troublesome dyskinesia in patients with advanced Parkinson's disease compared with immediate-release oral levodopa. Besides, this benefit was also associated with a significant reduction in off-time. On-time without troublesome dyskinesia in patients treated with LCIG was increased by 0.55 h compared with oral levodopa, and off-time was reduced by 1.19 h. These results were consistent with a recent systematic review (34), which also found that infusion of LCIG reduced off-time, increased on-time without increasing troublesome dyskinesias. There were no differences between groups in UPDRS part II activities of daily life (ADLs) and UPDRS part III motor scores. Similarly, LCIG failed to significantly improve UPDRS, PDQ-39 and Hoehn & Yahr scores. These data suggest that LCIG-treated patients may have approached maximum improvement on several efficacy measures (off-time and on-time without troublesome dyskinesia). The lack of significant further improvement may relate to the notable heterogeneity among these studies and the natural progression of the disease in these patients (24).

This meta-analysis also evaluated the tolerability and safety of LCIG in PD patients. The dropout rates for AEs were 7.6 and 9.1% in LCIG and control group (Figure 5B). Most subjects experienced at least 1 AE (85.0% in LCIG group vs. 73.0% in the control group) (Figure 6A), and most events were mild or moderate in severity. Severe AEs were reported by 11.4% in the LCIG group vs. 16.8% in the control group (Figure 6B). The pooled results showed that there were no clinically meaningful differences in the risk ratio of dropout, AEs and severe AEs between the continuing-LCIG and control patients. Thus, LCIG in Parkinson's disease seems safe and tolerated when compared with control conditions (oral medical treatment or levodopa-carbidopa immediate release). Significantly, Wirdefeldt et al. revealed that the safety issues mainly related to the intestinal infusion system (34). Thus, future studies may further enhance the safety of LCIG by improving the intestinal infusion system.

According to our knowledge, this is the first meta-analysis focusing on the efficacy and safety of LCIG for PD patients. After a comprehensive search, 8 prospective and retrospective studies with 384 PD patients were collected in our meta-analysis. Most included trials were of moderate to high quality. The selection bias was controlled since patients in two groups had a similar baseline condition, and the publication bias was acceptable in most comparisons except AE. However, only two studies used a double-blind design (12, 21), participants bias and rater bias could not be ignored in other studies. Among these included studies, Nyholm et al. also reported the pharmacokinetics changes after intestinal infusion of levodopa, and found that significantly lower variability in plasma levodopa levels can be achieved with the infusion of the stabilized carbidopa/levodopa suspension compared with oral sustained-release tablets (14). Jugel et al. emphasized that handling of LCIG should comprise screening and follow-up exams for polyneuropathy and determine B vitamin as well as folate levels in real time (18).

Cognitive impairment is one of the most important non-motor symptoms in PD, including PD-associated mild cognitive impairment (PD-MCI) and PD-associated dementia (PD-D). Although Valldeoriola et al. and Merola et al. found that LCIG treatment could not significantly improve the Mini-Mental State Examination (MMSE) scores (23, 27), a lot of studies argued that cognitive changes in the LCIG group could be related to a positive effect of L-dopa on some aspects of cognition, including executive function, language function, learning and recall, etc (27, 35, 36).

To ensure the study quality, the present meta-analysis only included trials with a control group. Actually, according to our search results, more studies focusing on LCIG for PD patients were self pre-and post-control observational studies and lack a control group. Most of them confirm the effectiveness and safety profile of LCIG in patients with advanced PD (11, 37–42), especially in the long-term follow-up (43–46). Zibetti and Kruger et al. also found the positive effects of LCIG infusion on sleep quality and quality of life in patients with advanced PD (47, 48). All included studies in this meta-analysis were performed in the USA and Europe, while Murata et al. added that LCIG was also efficacious and tolerable in Japanese, Taiwanese, and Korean advanced PD patients (42). Interestingly, apart from the efficacy and safety, LCIG is also cost-effective for PD patients (49).

Both deep brain stimulation (DBS) and LCIG are invasive therapies. Merola et al. demonstrated that DBS and LCIG showed comparable efficacy in activities of daily living and OFF time for PD patients (23). Besides, Valldeoriola et al. found that compared with patients receiving DBS, PD patients administrated with LCIG might significantly improve some specific neuropsychological functions such as learning, recognition, delayed recall, and visuospatial function (27). However, DBS might yield greater improvement in dyskinesia and lower long-term rate of complications than LCIG (23). Even so, some patients after DBS could also suffer from intolerable side effects, including dysarthria and stimulation-induced freezing of gait (50). For these patients, additional LCIG therapy may be helpful. In general, LCIG should be preferred to DBS for these older patients with more cognitive deficits (50).

There are several strengths in this meta-analysis. First, this systematic review and meta-analysis was designed and reported closely followed the standard PRISMA guidelines, which were comprehensible and concise for other peer researchers in this field. Second, this study is the first comprehensive meta-analysis to date focusing on the efficacy and safety of LCIG in subjects with PD. Another advantage of this study is that we summarized the outcomes as much as possible. The comprehensive reports of comparisons contribute to finding the shortages in these included studies. Moreover, the results of this meta-analysis have some practical implications for the researchers, clinicians and policymakers in this field. For example, future studies may further improve the LCIG system to ensure safety and maximize efficacy. The results also emboldened the clinicians and policymakers, and give them the confidence to conduct LCIG treatment in PD patients.

As with many systematic reviews and meta-analyses, our study has some limitations. First, the significant heterogeneity (*I*^2^ ≥ 50% or *p* ≤ 0.1) should be noted across these eligible original trials. The heterogeneity may be derived from the different study design (cohort studies and RCTs) or duration (from weeks to years), and cannot be ignored in some comparisons. Second, although most included studies had a moderate or high quality. The limited sample size may lead to false negative or positive conclusions. Moreover, patients of the control group in most studies were treated OMT (oral medical treatment) and LC-IR (levodopa-carbidopa immediate release), but Valldeoriola et al. compared LCIG with STN-DBS (subthalamic nucleus deep brain stimulation) in PD patients. Non-placebo control conditions may also underestimate the effects of LCIG.

## Conclusions

In conclusion, the present meta-analysis has provided robust evidence that LCIG improves off-time and on-time without troublesome dyskinesia. There were no significant differences in dropout ratio and AEs between LCIG and control groups, which suggested LCIG was relatively tolerated and safe. Therefore, LCIG may be a promising option for advanced PD patients with motor complications. More double-blind RCTs are needed in this field and future studies should pay more attention to unify its dosage and treatment duration.

## Author contributions

LW contributed to study design, data extraction, quality assessment, analysis and interpretation of data, and drafting the manuscript. JL contributed to data extraction, quality assessment, analysis and interpretation of data, and drafting the manuscript. JC conceiving the study, participating in study design and revising the article. All authors proofed and approved the submitted version of the article.

### Conflict of interest statement

The authors declare that the research was conducted in the absence of any commercial or financial relationships that could be construed as a potential conflict of interest.
